# Delay, Doubt and Dollars: Exploring Delay and Probability Discounting of Free and Paid HIV Vaccines among Sexual and Gender Minorities

**DOI:** 10.1007/s10461-026-05130-5

**Published:** 2026-04-22

**Authors:** Promise Tewogbola, Victoria Ose Bodunde, Oluwakamikun Faith Adekunle, Deborah Ojone Odaudu, Oluwatosin Tewogbola, Joy Ofuokwu-Oduniyi, Yueh-Ting Lee, Ryan N. Redner, Justin T. McDaniel, Jebaraj Asirvatham, Eric A. Jacobs

**Affiliations:** 1 School of Psychological and Behavioral Sciences, Southern Illinois University, Carbondale, IL 62901 USA; 2 PROMISE Labs Africa, Ibadan, Nigeria; 3 Department of Physiology, SIU School of Medicine, Southern Illinois University, Carbondale, IL 62901 USA; 4 Department of Sociology, Southern Illinois University, Carbondale, IL 62901 USA; 5 School of Health Sciences, Southern Illinois University, Carbondale, IL 62901 USA; 6 School of Agricultural Sciences, Southern Illinois University, Carbondale, IL 62901 USA

**Keywords:** HIV vaccine, Behavioral economics, Discounting, Delay discounting, Probability discounting, vacuna contra el VIH, economía conductual, descuento, descuento por demora, descuento por probabilidad

## Abstract

This study investigated how delays and uncertainties shape the perceived value of hypothetical HIV vaccines under different cost conditions among sexual and gender minorities (SGMs). In Experiment 1 (*N* = 260), participants chose between a 60% effective vaccine available immediately and a 99% effective vaccine with varying delays. Experiment 2 (*N* = 246) assessed choices between a 55% effective vaccine with certain availability and a 95% effective vaccine with uncertain availability. Participants were randomly assigned to free or $150 fee conditions. Hyperbolic discounting models effectively described delay (*R²* = 0.96–0.99) and probability discounting (*R²* = 0.95–0.99). Vaccine cost did not significantly affect discounting, while age and STI testing history did, with effects varying by delay versus probability context. Gay or bisexual identity, health insurance status, and political orientation additionally predicted probability discounting. The findings demonstrate the utility of behavioral economic models for quantifying HIV vaccine valuation prior to market availability.

## Resumen

Este estudio investigó cómo los retrasos y las incertidumbres influyen en el valor percibido de vacunas hipotéticas contra el VIH bajo diferentes condiciones de costo entre minorías sexuales y de género (MSG). En el Experimento 1 (*N* = 260), los participantes eligieron entre una vacuna con eficacia del 60% disponible de forma inmediata y una vacuna con eficacia del 99% con retrasos variables. El Experimento 2 (*N* = 246) evaluó elecciones entre una vacuna con eficacia del 55% con disponibilidad cierta y una con eficacia del 95% con disponibilidad incierta. Los participantes fueron asignados aleatoriamente a condiciones de acceso gratuito o pago de $150. Los modelos de descuento hiperbólico describieron eficazmente el descuento por demora (*R²* = 0.96–0.99) y el descuento por probabilidad (*R²* = 0.95–0.99). El costo de la vacuna no afectó significativamente el descuento, mientras que la edad y el historial de pruebas de ITS sí lo hicieron, con efectos que variaron según el contexto de demora o probabilidad. La identidad gay o bisexual, la cobertura de seguro médico y la orientación política predijeron adicionalmente el descuento por probabilidad. Los hallazgos demuestran la utilidad de los modelos económicos conductuales para cuantificar la valoración de vacunas contra el VIH antes de su disponibilidad en el mercado.

## Introduction

In the United States, HIV remains a public health issue, despite the country spending approximately 17% of its GDP on health-related expenses, according to the World Bank’s Global Health Expenditure database [1]. In impoverished urban areas, HIV prevalence mirrors that of several low-income countries experiencing widespread epidemics [2, 3]. Although substantial progress has been made in HIV prevention and treatment, persistent gaps remain. Approximately 15% of infected individuals remain undiagnosed, PrEP coverage reaches only about 36% of eligible individuals, and adherence challenges can contribute to continued transmission and, in some cases, drug-resistant strains [4–13]. These structural and behavioral barriers highlight the limitations of existing prevention strategies and underscore the need for complementary approaches.

Given these challenges, developing a safe and effective HIV vaccine could represent a transformative addition to current prevention efforts. Vaccines have historically been the most efficient method for preventing infectious diseases, as demonstrated with smallpox, polio, and COVID-19 [14–21]. Recent mRNA technology developments have reignited hope for an HIV vaccine, with the National Institute of Allergy and Infectious Diseases (NIAID) initiating clinical trials for three experimental HIV vaccines in March 2022 [22, 23]. However, the eventual success of an HIV vaccine program will not depend solely on vaccine development but also on understanding and addressing factors that influence vaccine acceptance among high-risk populations.

Established health behavior models such as the Health Belief Model [24], the Health Action Process Approach [25], and the Theory of Planned Behavior [26] have long informed our understanding of vaccine-related decisions. These frameworks identify cognitive and motivational antecedents of health behavior, including perceived susceptibility, severity, benefits, barriers, self-efficacy, and intentions [24–27]. However, they share a structural limitation in the context of HIV vaccine research: they were designed to explain decisions about health behaviors or interventions that already exist, where individuals can draw on prior experience, social norms, and concrete cost-benefit information. A market-ready HIV vaccine does not yet exist. This means that individuals have no direct experience with it, no social reference group that has accepted or refused it, and no established norms around which to orient their decisions. In this context, asking individuals to endorse attitudinal statements on Likert scales, as is common in this literature [28, 29], may elicit responses that reflect general vaccine sentiment or social desirability rather than how they would actually navigate real constraints of time, cost, and uncertainty during a future rollout.

Behavioral economics offers a complementary approach that addresses this limitation directly. Rather than measuring attitudes or intentions, discounting frameworks impose structured tradeoffs [30, 31] that mirror the kinds of constrained choices individuals will face during an actual vaccine rollout, where access may be delayed due to production or distribution timelines, or uncertain due to supply volatility and regulatory processes. Discounting tasks require repeated choices between alternatives that vary in timing, certainty, and magnitude, generating individual and group-level parameters that quantify how much vaccine value erodes under these conditions [32, 33]. Critically, these parameters can be estimated without requiring participants to have prior experience with the commodity in question, making discounting particularly well-suited to modeling decisions about a not-yet-available prevention technology. Additionally, discounting indices can be compared across subgroups and used to simulate uptake under hypothetical delivery scenarios, offering a prospective planning tool that attitudinal measures are not designed to provide. Thus, discounting frameworks allow prospective modeling of constrained behavioral choices for interventions that do not yet exist.

Two forms of discounting are especially pertinent to HIV vaccine acceptance: delay discounting and probability discounting. Delay discounting refers to the devaluation of a commodity as the time to its receipt increases. This phenomenon is observed across diverse contexts, from financial decisions where individuals may choose $500 today over $1,000 in one year, to dietary choices where people select immediately available fast food over preparing a healthier meal that requires additional time investment. In the vaccine context, delay discounting manifests if individuals prefer a less effective vaccine available immediately rather than waiting for a more effective vaccine promised in 12 months.

In contrast, probability discounting describes the devaluation of a commodity based on the uncertainty of its occurrence [34]. This pattern emerges in scenarios such as choosing a guaranteed $400 over a 50% probability of receiving $1,000, despite the latter having a higher expected value. Similarly, individuals may select readily available fast food over seeking healthier dining options with uncertain availability. Applied to vaccine decision-making, probability discounting occurs if individuals opt for a moderately effective vaccine with certain availability rather than waiting for a highly effective vaccine with only a 40% probability of approval and distribution.

To quantify how individuals evaluate delayed or uncertain access to HIV vaccines, several mathematical models of discounting can be applied. The most widely applied model is the hyperbolic discounting function, given as:

1 $${\mathrm{V}} = {\mathrm{A}}/\left( {{\mathrm{1}} + {\mathrm{kD}}} \right)$$

where *V* is the subjective value of the delayed reward, *A* is the amount of the delayed reward, *D* is the delay, and *k* represents the individual’s discounting rate. In some analyses, the parameter *k* serves as a key indicator - lower *k* values suggest a greater willingness to wait for the vaccine (self-control), while higher *k* values indicate a stronger preference for immediate alternatives (impulsivity). This model effectively captures how people often demonstrate time-inconsistent preferences, particularly relevant in healthcare decisions where benefits may be delayed [35]. Extended versions of this model introduce an additional parameter (*s*) to capture sensitivity to delay:

2 $${\mathrm{V}} = {\mathrm{A}}/\left( {{\mathrm{1}} + {\mathrm{kD}}} \right)^{{\mathrm{s}}}$$

3 $${\mathrm{V}} = {\mathrm{A}}/\left( {{\mathrm{1}} + {\mathrm{kD}}^{{\mathrm{s}}} } \right)$$

where a higher value of *s* indicates a greater sensitivity to delay.

Probability discounting follows a similar hyperbolic model, with subjective value *V* declining as the odds against accessing that commodity *(θ)* increase:

4 $${\mathrm{V}} = {\mathrm{A}}/\left( {{\mathrm{1}} + h\theta } \right)$$

where *h* represents the discounting rate with lower values indicating risk tolerance and higher values reflecting risk aversion [36]. Rachlin also describes the two-parameter hyperboloid forms of probability discounting [34]:

5 $${\mathrm{V}} = {\mathrm{A}}/\left( {{\mathrm{1}} + h\theta } \right)^{s}$$

6 $${\mathrm{V}} = {\mathrm{A}}/\left( {{\text{1 }} + h\theta ^{s} } \right)$$

In addition to model-based approaches, discounting severity can be summarized nonparametrically using the Area Under the Curve (AUC) method [37]:

7 $${\mathrm{AUC}} = \sum _{{i = 1}} \left( {x_{2} - x_{1} } \right)\,\,\left[ {\left( {y_{1} + y_{2} } \right)/{\mathrm{2}}} \right]$$

where *x*_*1*_ and *x*_*2*_ are normalized delay/probability values, and *y*_*1*_ and *y*_*2*_ are the corresponding normalized indifference points. Higher AUC values indicate less discounting (greater willingness to wait or accept uncertainty), whereas lower AUC values indicate more impulsive decision-making.

Discounting approaches have been applied to a range of health behaviors, including alcohol use [38], smoking [39], drug use [40], and COVID-19 vaccination decisions [41]. However, HIV vaccine acceptance presents a unique decision environment complicated by factors such as stigma, varied perceptions of risk, and existing structural impediments to healthcare. Prior research indicates that socioeconomic status correlates with variations in discounting parameters [42], and structural inequities such as housing instability, policy disparities, and unequal healthcare access are likely to influence health-related decision-making among racial minorities [43, 44]. Yet little is known about whether delay and probability discounting processes differ across racial and socioeconomic groups within Sexual and Gender Minority (SGM) populations, who bear a disproportionate burden of HIV. Discounting may therefore provide a useful framework for investigating how contextual and demographic factors influence the perceived value of an HIV vaccine.

Despite increasing focus on vaccine acceptance, research has rarely modeled HIV vaccine decisions as constrained behavioral choices under delay and uncertainty, particularly among SGMs, who are often underrepresented in vaccine decision-making research and can be difficult to engage through traditional sampling approaches [45]. The present study addresses this gap through three aims.

First, we examine whether delay and probability discounting curves generated through Simulated Purchase Tasks (SPTs) can characterize valuation of a prevention technology that is not yet available. SPTs are well-suited for this purpose: prior research indicates that discounting measures derived from hypothetical scenarios correlate with real-world behavior [46, 47], including predicting COVID-19 vaccine uptake nearly a year before vaccines entered clinical trials [48], and informing substance-use interventions [49, 50]. By modeling structured tradeoffs under delay and uncertainty, this approach extends beyond traditional vaccine hesitancy research, which typically examines established vaccines such as influenza or COVID-19 where participants already hold prior beliefs and experience.

Second, although contextual factors such as cost and availability are known to shape health decisions, their influence on HIV vaccine valuation under conditions of delay and uncertainty remains unclear. We therefore evaluate how vaccine cost (free versus $150), timing of availability (immediate versus delayed), and certainty of access (varying probabilities) influence willingness to accept vaccination. Finally, given the disproportionate HIV burden on SGMs and their underrepresentation in research, we examine how discounting of HIV vaccines varies based on participants’ personal and interpersonal characteristics, including age, gender, ethnicity, socioeconomic status, HIV risk behavior history, and religious and political orientations.

## Experiment 1

The purpose of Experiment 1 was to examine the impact of the cost of delayed HIV vaccines - available either for free or for a $150 fee - on how their value was discounted. We selected a single hypothetical out-of-pocket fee of $150 to represent a moderate financial barrier for experimental manipulation. At the time the study was designed, this value approximated the median price for vaccines in the United States [51], and was therefore used as a single experimental price estimate for a yet-unavailable HIV vaccine.

Participants were randomly divided into two groups: one with access to free HIV vaccines and the other with the option to purchase the vaccines for $150. In both conditions, participants made choices between a less effective vaccine that was available immediately and a more effective vaccine available after a series of increasingly longer delays.

### Participants

A sample of 393 participants was recruited through Reddit and organizations serving Sexual and Gender Minorities (SGMs) throughout the United States. The study protocol was approved by the Institutional Review Board (IRB) of a Midwestern state university. The survey, hosted on Qualtrics, presented participants with an informed consent document, and only those who provided consent could proceed with the study. Participation was voluntary and uncompensated.

### Procedure

The following measures were used in Experiment 1. Note that while demographic information and personal covariates are listed here for contextual relevance, they were collected after the completion of both Experiment 1 and Experiment 2.

Participants were permitted to partake in a simulated HIV Vaccine Delay Discounting Task (HVDDT) where they were provided with the following instructions:

Please read and consider the following. Imagine a situation in which two different HIV vaccines were available for XX for anybody who wanted to get immunized. HIV Vaccine A is 99% effective but only available in stock after a certain delay. HIV Vaccine B is 60% effective and is immediately available. Both vaccines are easy to use and take less than 5 min to administer. The following questions ask about whether or not you would get an HIV vaccine for XX, given a series of delays in receiving the vaccine itself. Remember that there are no “right” or “wrong” answers. Please respond honestly, as if you were actually in this scenario.

The value of XX characterized the experimental condition the participants were assigned to, whether HIV vaccines are available for free or for a $150 fee. Participants reported their preferences between an immediately available but less effective HIV Vaccine B and a more effective HIV Vaccine A, only available after a series of delays. The specific delays to assessing the more effective HIV Vaccine B were: 1 day, 1 week, 1 month, 6 months, 1 year, and 5 years. The selected delay intervals were chosen to reflect the range of delays typically used in human delay discounting research. Prior studies have examined discounting across a wide temporal range, from immediate or short delays extending to one year [52], through multi-year intervals of up to a decade [32]. The intervals used in the present study were selected to reflect this established range while remaining within timeframes participants could realistically envision. Such durations are also plausible in the context of HIV vaccine development and rollout, where extended timelines may result from multi-phase clinical trials, manufacturing scale-up, phased eligibility prioritization, and distribution constraints.

The 60% effectiveness level was selected to represent a moderately effective vaccine within ranges observed for existing vaccines. Seasonal influenza vaccines, for example, commonly demonstrate effectiveness between 40% and 60% when well-matched to circulating strains [53]. This value also created a clear contrast with the 99% option, requiring participants to weigh immediacy against higher protection.

#### Risk Behavior Assessment

Participants responded to a series of dichotomous yes-no questions designed to assess their risk of contracting HIV. These include previous HIV testing, recent STI testing (excluding HIV), PrEP usage in the past year, history of injecting non-prescribed drugs, condomless sex in the past year, and non-prescribed drug use in the past 30 days.

#### Subjective HIV Risk Perception

Participants rated their perceived risk of HIV infection on a VAS ranging from 0 (“Very unlikely”) to 100 (“Very likely”) in response to the question: “*What is your gut feeling about how likely you are to get infected with HIV?*”

#### Trust

Trust in scientific research, medical professionals, and the US government were each assessed on a 100-point scale (0 = no trust at all; 100 = a great deal of trust).

#### Political Views

Participants rated their political views on a VAS ranging from 0 *(*“*Very conservative/not liberal at all*”) to 100 *(*“*Very liberal/not conservative at all*”).

#### Discrimination Experience

Participants indicated (*Yes/No*) whether they had experienced discrimination based on race, gender, or sexual orientation.

#### Vaccination History

Participants reported (*Yes/No*) whether they had received any vaccine (e.g., flu, COVID-19, etc.) in the past 3 years.

#### Health Insurance Coverage

Participants responded “*Yes*” or “*No*” to the question: “*Do you currently have health insurance or health care coverage?*”

#### Demographics

Participants provided comprehensive demographic information as part of the study. They reported their assigned gender at birth, choosing from options including ‘*Male’*,*‘Female’*,*‘Other’*, or ‘*Prefer not to say’*. Regarding their sexual partners in the past year, participants selected *from ‘Men only’*,*‘Women only’*,*‘Both men and women’*,* ‘I have not had sex’*, or ‘*Other’.* Age was reported in years. For race and ethnicity, participants self-identified their racial identity, with options including ‘*Black/African American’*,*‘Hispanic/Latino’*,*‘Indigenous Peoples’*,*‘Middle Eastern or North African’*,*‘Pacific Islander’*,*‘White or Caucasian’*,*‘Asian’*, or ‘*Other’*. Marital status was categorized as ‘*Married’*,*‘Living together as married’*,*‘Separated’*,*‘Divorced’*,*‘Widowed’*, or ‘*Never married’*. Total annual household income was reported in brackets: ‘*9*,*999’‘$10*,*000-$24*,*999’‘$30*,*000-$49*,*999’‘$50*,*000-$74*,*999’‘$75*,*000-$99*,*999’‘$100*,*000-$149*,*999’*, or ‘*$150*,*000+’*. Educational attainment was assessed by highest level completed, ranging from ‘*Never attended school’* to ‘*Any post-graduate studies’*, with intermediate options *including ‘Grade 12 or GED’*,*‘Some college*,*Associate’s Degree*,*or Technical Degree’*,*and ‘Bachelor’s Degree’*. Participants also described their residential area as ‘*An inner-city area’*,*‘A suburban area’*,*‘A town’*,*‘A village’*, or ‘*Rural or countryside’*.

### Statistical Analyses

The two-stage approach described by Kaplan and colleagues [54] was used to evaluate the discounting of both delayed HIV vaccines and monetary rewards. First, individual-specific discounting rates were estimated using the AUC method. The process involves normalizing both delays (*D*) and the value of the more effective HIV vaccine (*A*), such that they span from 0 to 1. AUC is then determined by summing the areas of the trapezoids formed between each interval, calculated using the following:

where *x*_*1*_ represents the normalized smaller delay to accessing the more effective HIV vaccine, *x*_*2*_ is the normalized larger delay, and *y*_*1*_ and *y*_*2*_ are the corresponding normalized valuations of the more effective HIV vaccine. That said, the one-parameter hyperbolic discounting model was also used to describe delay discounting at the aggregate level:

where *V* is the subjective value of the delayed vaccine; *A* is the amount or magnitude of the delayed vaccine; *D* is the delay in assessing the more effective vaccine; and, a parameter, *k*, reflects the discounting rate.

The primary outcome measures of interest in this study were the AUC values for discounting delayed HIV vaccines, which were obtained from the first stage of data analysis. Due to the non-normal distribution of AUC values, a logarithmic transformation was applied before proceeding to the second stage of analysis. This subsequent phase involved a multiple linear regression analysis conducted through three separate models. The first model used the experimental conditions as predictors to assess their direct impact on discounting AUC values. The second model examined the influence of demographic variables and other personal characteristics of participants. The third model combined both sets of predictors, offering a more holistic view of the factors affecting the discounting indices.

Several categorical variables were included in the analysis, each assigned specific coding schemes. Sex at birth was classified as ‘Male’ (coded as ‘1’) versus all other categories (coded as ‘0’). The biological sex of sexual partners was categorized as exclusively ‘Men’ or ‘Both men and women’ (coded as ‘1’), with all other responses grouped together. Ethnicity was dichotomized as ‘White’ (coded as ‘1’) versus other ethnicities. Socioeconomic status was based on household income, with those earning below $75,000 coded as ‘1’ and those earning above this threshold coded as ‘0’. Marital status was distinguished between individuals who were ‘Married’ or ‘Living together as married’ (coded as ‘1’) and those with other relationship statuses. Educational attainment was grouped into ‘Some college or higher’ (coded as ‘1’) versus lower levels of education. Residential location was categorized as ‘Inner city’ or ‘Suburban’ (coded as ‘1’), with all other areas classified separately.

For behavioral and health-related variables, negative responses (’No’) served as reference categories throughout the analysis. This also applied to HIV testing history, other STI screening, PrEP usage, injection drug use, experiences of racial discrimination, health insurance status, unprotected sexual activity, substance use, and vaccination history. Continuous variables retained their original scaling, including age, trust (in science, in government, and in medical professionals), religiosity, political liberalism, and perceived HIV risk, where higher scores indicated greater levels of each measure.

### Results

Incomplete or missing entries were identified and excluded from further analysis. As a result, 133 participants (33.84% of the dataset) were removed based on this exclusion criterion. Consequently, data from 260 participants met the inclusion criteria and were retained for the second stage of analysis. A summary of the demographic characteristics of the final sample is presented in Table 1.

**Fig. 1:**
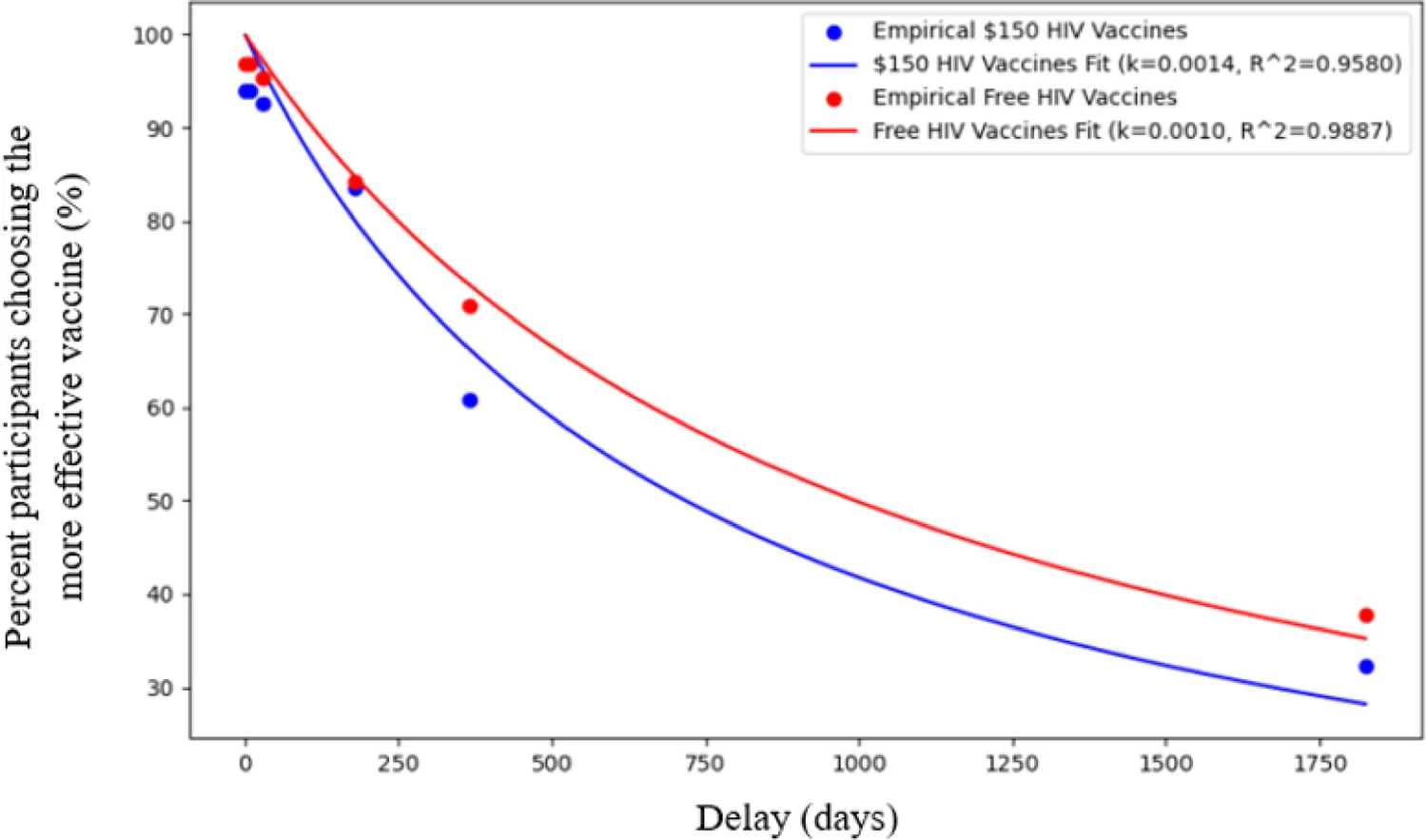
Aggregate delay discounting of a more effective but delayed HIV vaccines available for free vs. for $150. Plotted are group discounting curves for a more effective but delayed HIV vaccine available for free (red) and for a $150 fee (blue). Curves were plotted using the one-parameter hyperbolic discounting function (color figure online)

**Fig. 2:**
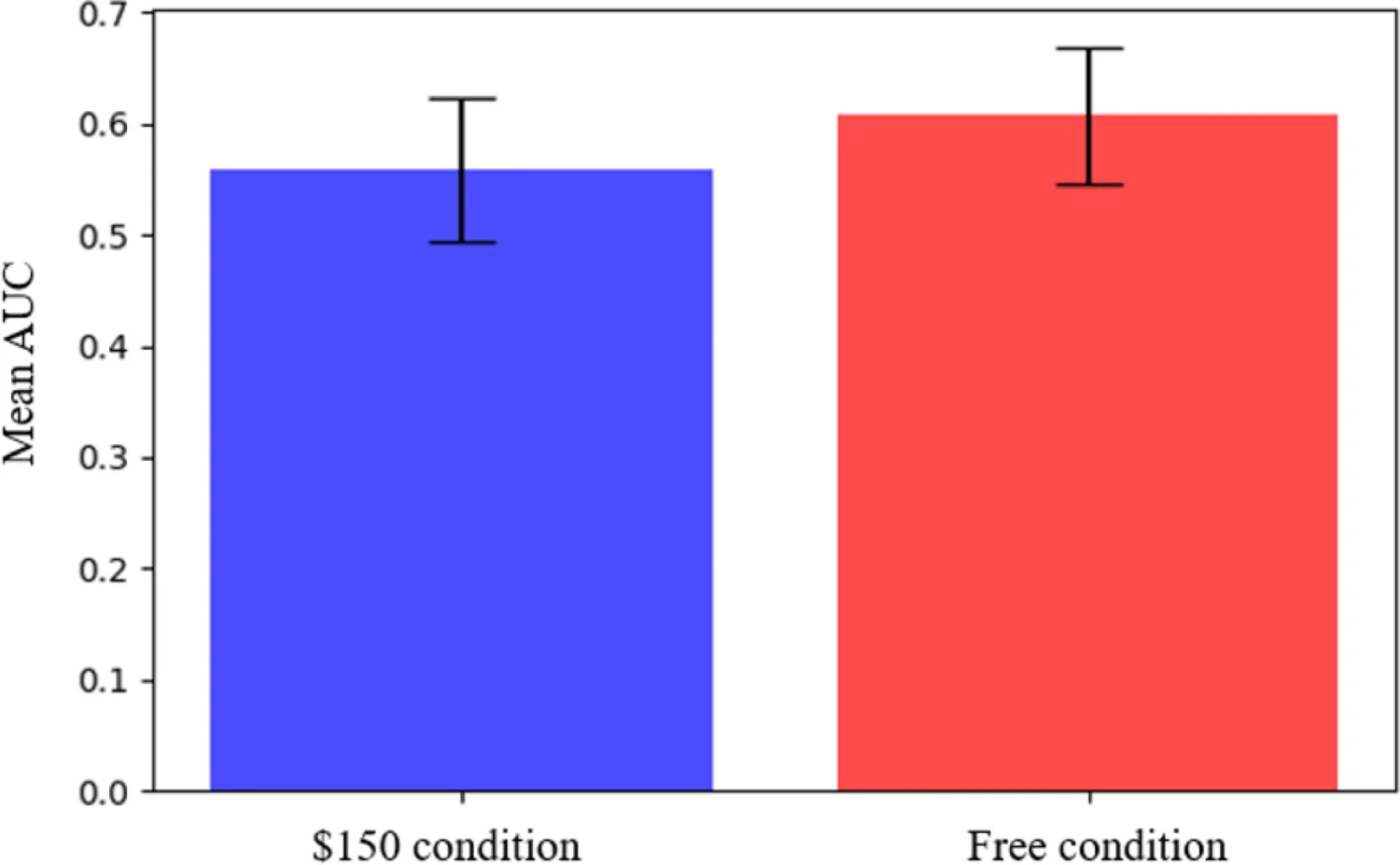
Bar charts of mean aucs for a more effective but delayed HIV vaccines available for Free vs. for $150. The bars represent the group mean AUCs for a more effective, delayed HIV vaccines available for free (red) and for a $150 fee (blue). Error bars indicate 95% confidence intervals (color figure online)

The hyperbolic discounting function effectively modeled delay discounting of HIV vaccines in the contexts of both the free and the $150 fee conditions, demonstrating high accuracy. This is evidenced by the substantial R-squared values obtained for the two experimental groups (Free: *R*^*2*^ = 0.99; $150 fee: *R*^*2*^ = 0.96). The computed aggregate *k*-value was 0.0010 for the free condition and 0.0014 for the $150 fee condition. Figure 1 displays the optimally fitted hyperbolic discounting function for each of these conditions. Furthermore, no significant differences were found in the AUC between the two experimental groups (Free: *M =* 0.60, *SD =* 0.35; $150 fee: *M =* 0.53, *SD =* 0.38; *t*(258) = 1.52, *p* =.13). The comparative AUCs for both conditions are depicted in a bar chart in Fig. 2. Moreover, when examining the correlation between the log-transformed AUCs from the Monetary Delay Discounting Task and the HIV Vaccine Delay Discounting Task, it was found to be not significant, *r*(205) = 0.02, *p* =.75. Figure 1 displays the aggregate discounting curves for delayed HIV vaccines and delayed monetary rewards.

Results from three regression models investigating the determinants of variability in the log-transformed delay discounting AUCs are presented in Table 2. The first model, which considered the experimental context of HIV vaccines being available either for free or at a $150 fee as predictors, found the effect size of this context on delay discounting for HIV vaccines was small (*R*^*2*^ = 0.004). There were also no significant differences between the reference comparison group (vaccines for free) and the other condition (vaccine for $150), even though the mean AUC was greater for the reference group. On the other hand, the second model that focused on demographics and other personal characteristics of participants offered more explanatory power (*R*^*2*^ = 0.159). Within this model, the significant predictors for delay discounting included a history of undergoing screening tests for STIs other than HIV (*β* = −0.19, *p* <.05), and age (*β* = 0.01, *p* <.05). The third model, integrating both the experimental context and personal factors, demonstrated a marginal increase in explanatory power over the second model (*R*^*2*^ = 0.16).

**Table 1:** Characteristics of participants meeting inclusion criteria for experiment 1 (*N* = 260)

	Mean (SD); *N* (%)
Vaccine cost	
Free	127 (48.85%)
$150	133 (51.15%)
Demographics and personal characteristics	
Age	29.37 (7.86)
Born male	80 (28.07%)^a^
Bisexual/Had sex with other men	100 (35.09%)
White	144 (50.18%)
Married/Living together as married	55 (19.3%)
Household income <$75,000	118 (41.4%)
At least some college education	180 (63.16%)
Done HIV test	134 (47.02%)
Done other STI test	89 (31.23%)
Taken PrEP	39 (13.68%)
Injected drugs	15 (5.26%)
Been racially discriminated	142 (49.82%)
Has health insurance	183 (64.21%)
Had unprotected sex	117 (41.05%)
Consumed alcohol	140 (49.12%)
Consumed other drugs	76 (26.67%)
Taken other vaccine	175 (61.4%)
Inner city/Suburban residency	145 (50.88%)
Trust in science	86.15 (19.19)
Trust in medical professionals	76.87 (23.27)
Trust in government	46.34 (28.87)
Liberalism	84.49 (22.43)
Religiosity	28.39 (29.78)
Perceived risk of HIV infection	19.80 (24.19)

^a^Participants were first asked “Sex assigned at birth” and then “Are you transgender?” Of those assigned male at birth (*n* = 80), 21 identified as transgender. A separate gender identity item was not collected; only sex assigned at birth and transgender status were assessed

For discrete variables, the number of participants and percentage of sample are included. For continuous variables, mean and SD are included

**Table 2:** Regression Results Showing Effect of the Experimental Context of HIV Vaccines Available either for Free or at a $150 Fee and Personal Characteristics on Delay Discounting AUCs

Predictor	log AUC		
	β	*p*	*R* ^2^
Model 1: Experimental context			
Vaccines for free (reference)	**−0.37**	**< 0.001** ^***^	0.004
Vaccines for $150 fee	−0.06	0.31	
Model 2: Personal characteristics			
Age	**0.01**	**0.02** ^*^	0.159
Born male	−0.02	0.85	
Bisexual/Had sex with other men	0.09	0.28	
White	−0.11	0.21	
Married/Living together as married	−0.08	0.40	
Household income <$75,000	0.09	0.26	
Education			
Done HIV test	0.13	0.13	
Done other STI test	**−0.19**	**0.03** ^*^	
Taken PrEP	−0.09	0.46	
Injected drugs	−0.31	0.08	
Been racially discriminated	0.14	0.12	
Has health insurance	−0.17	0.28	
Had unprotected sex	0.01	0.91	
Consumed alcohol	−0.01	0.90	
Consumed other drugs	0.05	0.51	
Taken other vaccine	−0.04	0.80	
Inner city/Suburban residency	0.09	0.30	
Trust in science	0.0002	0.94	
Trust in medical professionals	0.0001	0.54	
Trust in government	0.0005	0.78	
Liberalism	−0.003	0.21	
Religiosity	0.002	0.24	
Perceived risk of HIV infection	0.002	0.30	
Model 3: Experimental context and personal characteristics			
Predictors in Model 1 and 2	YES^c^	YES^c^	0.160

Model 1 evaluated the effect of cost on discounting AUC values of delayed HIV vaccines while Model 2 assessed the impact of participant covariates. Statistically significant values are bolded

*p <.05. **p <.01. ***p <.001

## Experiment 2

The objective of Experiment 2 was to assess how vaccines are discounted under increasing levels of uncertainty when the vaccines are available for free or for $150, depending upon the condition. Similar to Experiment 1, participants were randomly assigned into two groups: one had the opportunity to receive an HIV vaccine at no cost, while the other had the option to purchase the vaccine for $150. Participants in each group completed a series of choices between a less effective HIV vaccine that was readily available and a more effective vaccine that was probabilistically available. The probability of obtaining the more effective vaccine varied across choices and ranged from 10% to 90%.

### Participants

Upon completing Experiment 1, participants transitioned directly into Experiment 2.

### Procedure

Participants were permitted to partake in a simulated HIV Vaccine Probability Discounting Task (HVPDT) where they were provided with the following instructions:

Please read and consider the following. Imagine a situation in which two HIV vaccines are available for XX only at health centers, hospitals, and clinics in your city. HIV Vaccine C is 55% effective and is always available. HIV Vaccine D is 95% effective but is not always available due to high demand. You have the same income and savings as you do now, and vaccines are the only available options for protecting against HIV. You are aware of the risks associated with not getting vaccinated, and you understand the benefits of receiving the vaccine. The following questions ask about the likelihood of you booking an appointment to receive the vaccine, given various scenarios. Remember that there are no “right” or “wrong” answers. Please respond honestly, as if you were actually in this scenario.

The value of XX varied by the experimental condition that the participants were assigned to, whether HIV vaccines were available for free or for a $150 fee. Participants reported their preferences between a less effective but always available HIV vaccine C and an HIV vaccine D that was 95% effective but had varying probabilities of availability: 10%, 25%, 50%, 75%, and 90%. The 55% effectiveness level was selected to represent a moderately effective vaccine and to create a clear contrast with the 95% alternative, consistent with effectiveness levels reported for established vaccines such as seasonal influenza vaccines, which often fall within the 40–60% range when well-matched to circulating strains [53]. This ensured that participants were choosing between moderate effectiveness and substantially higher protection.

Please note that demographic information and additional personal covariates were collected after the completion of this experiment, as described in the Measures section of Experiment 1.

### Statistical Analyses

Prior to any data analyses, the five likelihood values were converted to odds-against values using the following equation:

8 $$\theta = \left( {{\mathrm{1}} - p} \right)/p$$

where θ is the odds against accessing the more effective HIV vaccine and *p* is the probability of not accessing the vaccine. The resulting values were: 0.11, 0.33, 1, 3 and 9. Consequently, the two-stage approach described in Experiment 1 was used to evaluate the discounting of both uncertain HIV vaccines and monetary rewards. Individual-specific discounting rates were estimated using the AUC method by normalizing both odds-against values (θ) and the value of the more effective HIV vaccine (*A*) so that they spanned from 0 to 1. AUC values were then estimated using the following:

where *V* is the subjective value of the more effective HIV vaccine; *A* is the amount or magnitude of the vaccine; *θ* is the odds against assessing the more effective vaccine; a parameter, *h*, reflects the discounting rate, and another parameter, *s*, depicts sensitivity to increasing odds-against receiving the more effective vaccine.

As was the case in Experiment 1, the primary outcome variable was the AUC values for discounting uncertain HIV vaccines. Due to the non-normal distribution of AUC values, a logarithmic transformation was applied before proceeding to the second stage of analysis. This subsequent phase involved a multiple linear regression analysis conducted through three separate models. The first model used the experimental conditions as predictors to assess their direct impact on discounting AUC values. The second model examined the influence of demographic variables and other personal characteristics of participants. The third model combined both sets of predictors.

### Results

Incomplete or missing entries were excluded, leading to the removal of 147 individuals, constituting 37.4% of the initial dataset. Consequently, data from 246 participants were carried forward for further analysis. Table 3 provides a summary of the demographic characteristics of study participants.

**Fig. 3:**
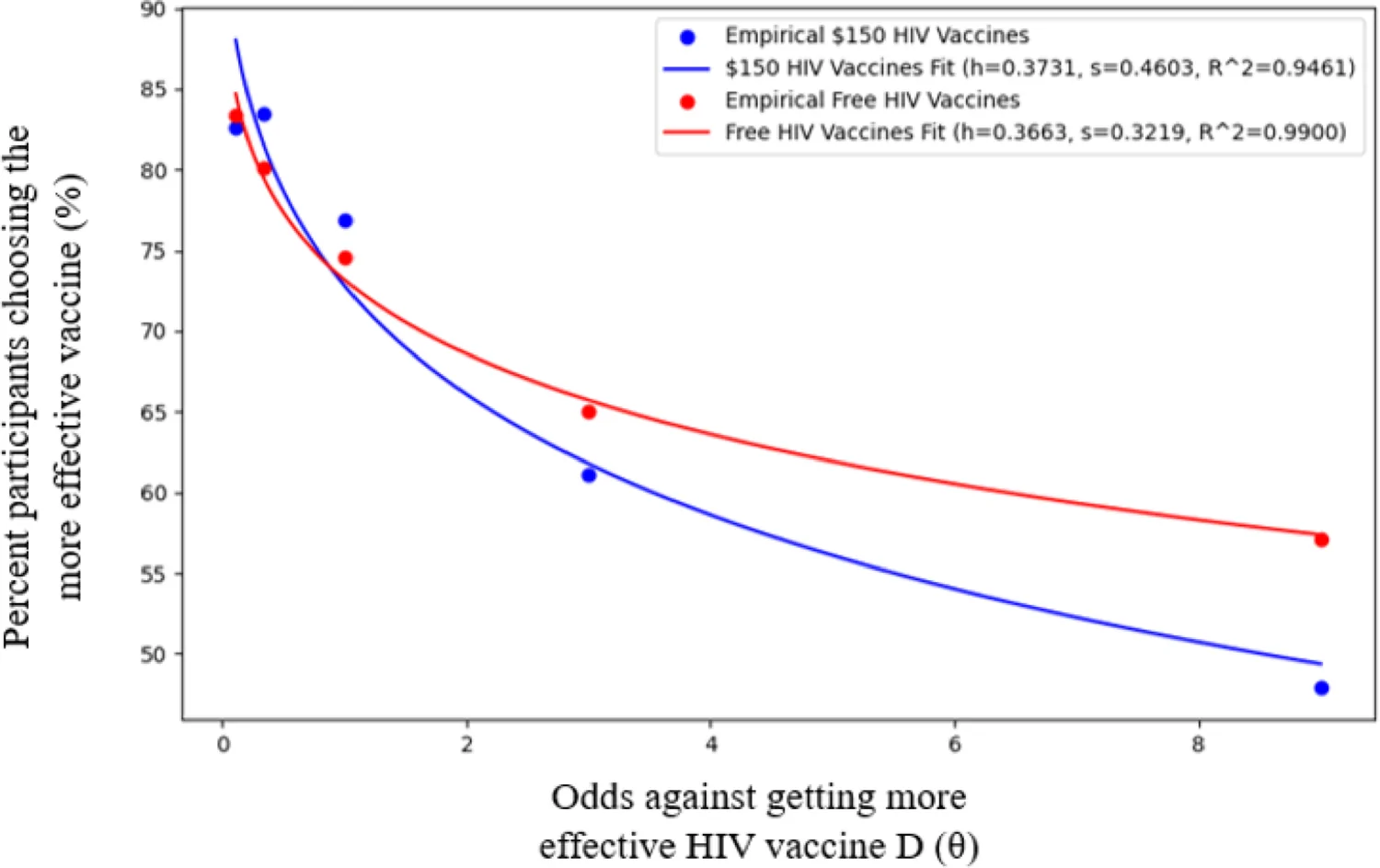
Aggregate probability discounting of a more effective but uncertain HIV vaccine available for free vs. Plotted are group discounting curves for a more effective but uncertain HIV vaccines available for free (red) and for a $150 fee (blue). Curves were plotted using a two-parameter hyperboloid model including a nonlinear scaling parameter on odds against getting the more effective vaccine. Note how at small odds against accessing the more effective vaccine, discounting of free vaccines is more pronounced than that of the $150 vaccines. At larger odds, however, discounting of the $150 vaccines is more than that of the free vaccines (color figure online)

**Fig. 4:**
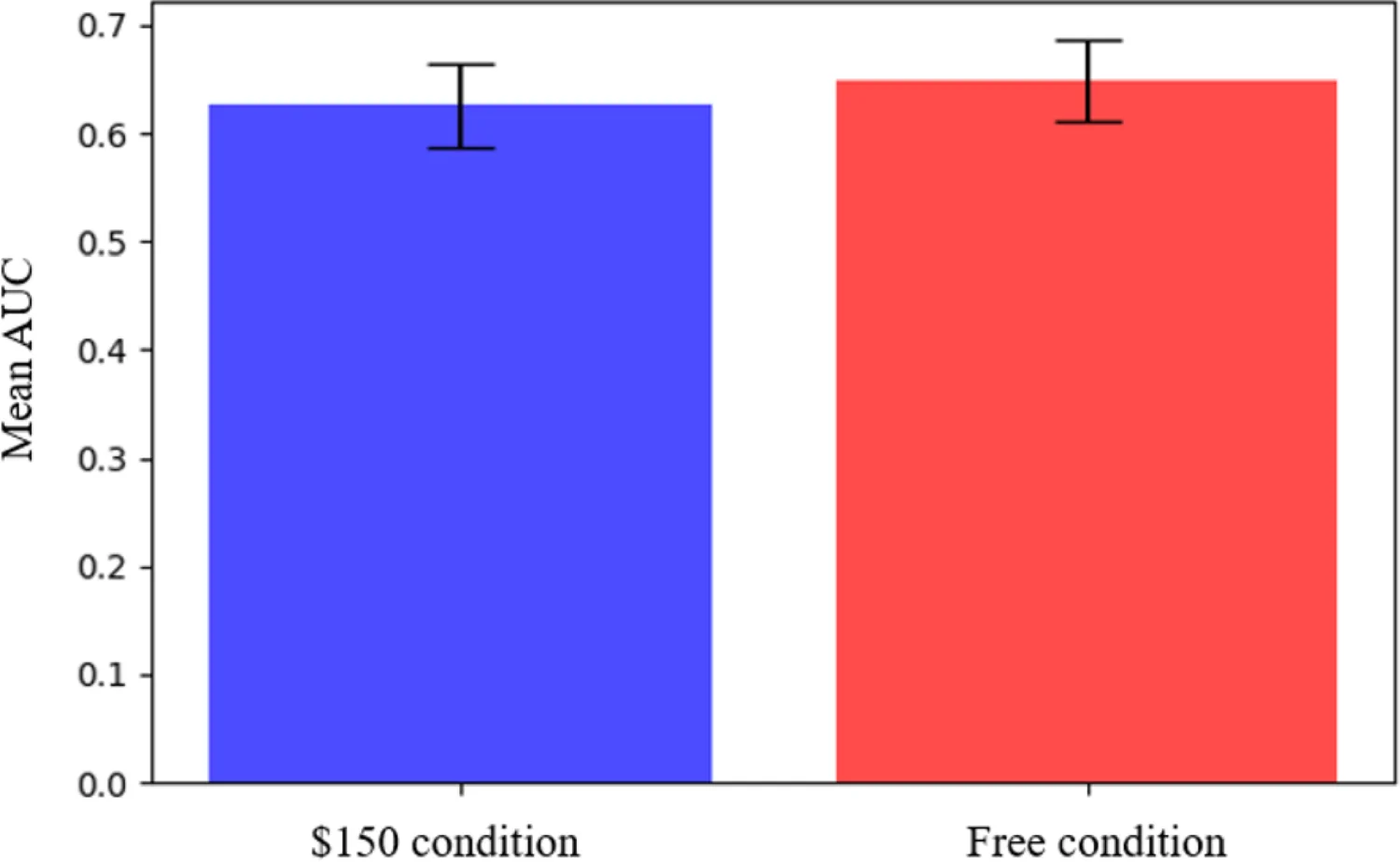
Bar charts of mean probability discounting AUCs for a more effective but uncertain HIV vaccine available for a fee vs for Free. The bars represent the group mean AUCs for a more effective but uncertain HIV vaccine available for free (red) and for a $150 fee (blue). Error bars indicate 95% confidence intervals (color figure online)

 The hyperbolic discounting function was employed to model the probability discounting of HIV vaccines under both free and $150 fee conditions as shown by the R-squared values for the two experimental conditions (Free: *R*^*2*^ = 0.99; $150 fee: *R*^*2*^ = 0.95). The aggregate *h*-value calculated was 0.3663 for the free vaccine condition and 0.3731 for the vaccine with the $150 fee. Figure 3 illustrates the best-fitting hyperbolic discounting function for each condition. Additionally, there were no significant differences in the AUC between the two groups (Free: *M =* 0.64, *SD =* 0.40; $150 fee: *M =* 0.60, *SD =* 0.39; *t*(244) = −0.75, *p* =.45), as shown in a bar chart in Fig. 4. Furthermore, upon investigating the correlation between the log-transformed AUCs from the Monetary Probability Discounting Task and the HIV Vaccine Probability Discounting Task, it was determined to be insignificant, with a correlation coefficient of *r*(185) = −0.07 and a p-value of 0.34. Figure 3 displays the aggregate discounting curves for uncertain HIV vaccines and probabilistic monetary rewards. At the aggregate level, Fig. 3 illustrates how as the odds against obtaining a more effective vaccine increase, the demand for the less effective vaccine rises accordingly, indicating a substitutionary relationship between the two vaccines. This also shows that the odds against receiving the more effective vaccine can function similarly to a cost/price variable in impacting vaccine demand.

Table 4 presents the outcomes of three regression models that explored what influences the variation in the log-transformed probability discounting AUCs. The initial model, which used the experimental condition of HIV vaccine costs (either free or at a cost of $150) as predictors, indicated a small effect of this context on probability discounting (*R*^*2*^ = 0.005). There were also no significant differences between the reference comparison group (vaccines for free) and the other condition (vaccine for $150), even though the mean AUC was greater for the reference group. In the second model, the significant predictors for probability discounting included identifying as gay or bisexual (*β* = −0.15, *p* =.05), having health insurance (*β* = 0.33, *p* <.05), a history of undergoing screening tests for STIs and other than HIV (*β* = 0.18, *p* <.05), being politically left-leaning (*β* = −0.004, *p* =.05) and age (*β* = 0.01, *p* <.05). The third model, which combined the experimental context with personal factors, showed a slight improvement in explanatory power compared to the second model (*R*^*2*^ = 0.198).

**Table 3:** Characteristics of participants meeting inclusion criteria for experiment 2 (*N* = 246)

	Mean (SD); *N* (%)
Vaccine cost	
Free	126 (51.22%)
$150	120 (48.78%)
Demographics and personal characteristics	
Age	29.37 (7.86)
Born male	80 (28.07%)^a^
Bisexual/Had sex with other men	100 (35.09%)
White	144 (50.18%)
Married/Living together as married	55 (19.3%)
Household income <$75,000	118 (41.4%)
At least some college education	180 (63.16%)
Done HIV test	134 (47.02%)
Done other STI test	89 (31.23%)
Taken PrEP	39 (13.68%)
Injected drugs	15 (5.26%)
Been racially discriminated	142 (49.82%)
Has health insurance	183 (64.21%)
Had unprotected sex	117 (41.05%)
Consumed alcohol	140 (49.12%)
Consumed other drugs	76 (26.67%)
Taken other vaccine	175 (61.4%)
Inner city/Suburban residency	145 (50.88%)
Trust in science	86.15 (19.19)
Trust in medical professionals	76.87 (23.27)
Trust in government	46.34 (28.87)
Liberalism	84.49 (22.43)
Religiosity	28.39 (29.78)
Perceived risk of HIV infection	19.80 (24.19)

^a^Participants were first asked “Sex assigned at birth” and then “Are you transgender?” Of those assigned male at birth (*n* = 80), 21 identified as transgender. A separate gender identity item was not collected; only sex assigned at birth and transgender status were assessed.

For discrete variables, the number of participants and percentage of sample are included. For continuous variables, mean and SD are included

**Table 4:** Regression results showing effect of the experimental context of HIV vaccines available either for free or at a $150 fee and personal characteristics on probability discounting AUCs

Predictor	log AUC		
	β	*p*	*R* ^2^
Model 1: Experimental context			
Vaccines for free (reference)	−**0.27**	**< 0.001** ^*^	0.005
Vaccines for $150	−0.07	0.28	
Model 2: Personal characteristics			
Age	**0.01**	0.02*	0.197
Born male	0.03	0.74	
Bisexual/Had sex with other men	−**0.15**	0.05*	
White	−0.02	0.82	
Married/Living together as married	−0.08	0.34	
Household income <$75,000	−0.01	0.94	
Education	0.11	0.46	
Done HIV test	−0.01	0.91	
Done other STI test	**0.18**	0.03*	
Taken PrEP	0.11	0.37	
Injected drugs	0.05	0.75	
Been racially discriminated	−0.02	0.85	
Has health insurance	**0.33**	0.02*	
Had unprotected sex	0.04	0.67	
Consumed alcohol	−0.13	0.12	
Consumed other drugs	0.02	0.78	
Taken other vaccine	−0.04	0.79	
Inner city/Suburban residency	0.11	0.17	
Trust in science	0.004	0.17	
Trust in medical professionals	−0.003	0.22	
Trust in government	−0.002	0.18	
Liberalism	−**0.004**	0.05*	
Religiosity	−0.0002	0.90	
Perceived risk of HIV infection	−0.001	0.48	
Model 3: Experimental context and personal characteristics			
Predictors in Model 1 and 2	YES ^c^	YES ^c^	0.198

Model 1 evaluated the effect of cost on discounting AUC values of delayed HIV vaccines while Model 2 assessed the impact of participant covariates. Statistically significant values are bolded

*p <.05. **p <.01. ***p <.001

## General Discussion and Conclusion

This study examined how delay and uncertainty shape the perceived value of HIV vaccines among SGMs, and how individual characteristics and vaccine cost moderate those effects. Across both experiments, the hyperbolic discounting function provided an excellent fit to aggregate data, confirming that the perceived value of a more effective HIV vaccine declines systematically as delays lengthen or as the probability of access decreases. This indicates that HIV vaccine acceptance can be conceptualized not merely as an attitudinal stance, but as a value allocation problem under constraint. Vaccine cost ($150 versus free) had a small and non-significant effect on discounting in both experiments, suggesting that within this price range, cost alone does not substantially alter how individuals weigh delayed or uncertain vaccine access. Personal characteristics, particularly age, STI testing history, health insurance status, sexual orientation, and political orientation, were more consistent predictors of discounting.

The average AUCs were modestly lower in the $150 condition than in the free condition across both experiments, indicating slightly steeper discounting when a cost was attached. If the $150 cost was framed psychologically as a loss, the small and non-significant differences observed here diverge from findings that probabilistic gains are discounted more steeply than losses [55, 56]. That said, since the effect size of the experimental context was small, and group differences in AUC were non-significant, strong conclusions about the role of cost are not warranted from this manipulation alone.

Age emerged as a consistent predictor across both experiments, with older participants demonstrating higher AUC values, indicating less discounting and greater willingness to tolerate delay and uncertainty in accessing a more effective vaccine. This result aligns with other studies [57, 58] that found delay discounting decreases with age, which may reflect reduced impulsivity, accumulated experience with delayed outcomes, or the moderating role of income on temporal decision-making [59]. However, this contrasts with Mok et al. [60], who reported no relationship between age and delay discounting in monetary decisions, and Seaman et al. [61], who observed higher rates of discounting for health benefits among older people, suggesting that domain specificity matters and that HIV vaccine decisions may engage different considerations than general health or monetary rewards.

A history of STI testing predicted discounting across both experiments, although the direction of effects differed. In the delay model, prior STI testing predicted lower AUC values, indicating steeper discounting of delayed vaccine access, whereas in the probability discounting model, it predicted higher probability discounting AUC values, indicating greater tolerance for uncertain access. This pattern may suggest that individuals with STI testing experience respond differently depending on the nature of the barrier: when it is uncertainty, they appear more tolerant, consistent with findings that perceived susceptibility predicts STI testing and risk-responsive behavior [62–65] but when the barrier is time, they appear less willing to wait.

Health insurance was associated with higher probability discounting AUCs, suggesting that insured participants were more tolerant of uncertainty in accessing a more effective vaccine. This mirrors findings that insured individuals are more likely to pursue vaccination generally [66, 67], and is consistent with Finkelstein and colleagues’ [68] framing of health insurance as a psychological and financial buffer that reduces the perceived cost of health-seeking under uncertainty. The moral hazard literature offers a complementary explanation: reduced financial exposure may lower the threshold at which individuals are willing to pursue healthcare, including novel vaccines with uncertain availability [69].

In the probability discounting analysis, identifying as gay or bisexual was linked with lower AUCs, indicating that these participants discounted uncertain HIV vaccine access more steeply. In other words, they placed greater value on reliable access to the more effective vaccine and were less willing to accept uncertainty as a barrier. This finding warrants careful interpretation given that gay and bisexual men bear the greatest burden of HIV in the United States. One possible explanation is heightened perceived vulnerability to HIV infection [70]: prior research has shown that among men who have sex with men (MSM), greater sexual risk behavior predicts acceptance of preventive vaccines [71], and that perceived risk of infection is associated with vaccine uptake [72]. From this perspective, steeper discounting of uncertain vaccine access may reflect how much this group values reliable HIV prevention, which makes the certain but less effective alternative comparatively more acceptable when the better option is probabilistic. Structural and experiential factors may also play a role. Newman and colleagues [73] documented barriers to novel vaccine uptake among racialized SGMs including systemic racism, homophobia and transphobia in healthcare institutions, digital access barriers, and the absence of culturally tailored information, which are all experiences that may erode tolerance for the added barrier of uncertain access. Additionally, familiarity with established HIV prevention strategies such as PrEP may shape expectations for consistent access to prevention tools [74], making probabilistic vaccine availability less acceptable by comparison.

Participants with a left-leaning political orientation showed modestly lower probability discounting AUCs compared to other participants, suggesting they were less tolerant of uncertainties in accessing HIV vaccines. This is consistent with research showing that left-leaning individuals express greater support for vaccination and vaccine mandates [75, 76], which Lin et al. [77] attribute partly to stronger institutional trust and greater concern for public health protection. This may be what we have observed in the present study, where left-leaning participants demonstrated lower tolerance for uncertainty in vaccine access, potentially reflecting values that prioritize health equity and access to healthcare [78].

Several other person-level factors, including race, HIV testing history, PrEP use, alcohol consumption, urban residency, and trust in medical professionals, did not significantly predict discounting in either experiment. These null findings should be interpreted with caution. Our sample was recruited through online platforms and SGM-serving organizations, which likely limited socioeconomic and racial diversity. Prior research suggests discounting parameters do vary by race and socioeconomic status [42–44], and the absence of effects here may reflect restricted variability in our sample rather than a true absence of structural influences on HIV vaccine decision-making. Studies using more diverse recruitment strategies are needed to test this more rigorously.

These findings carry implications for HIV vaccine implementation planning. The consistent role of age, insurance status, and sexual orientation suggests that discounting-based frameworks can identify subgroups likely to respond differently to vaccine delivery structures. Younger participants, uninsured individuals, and gay or bisexual participants demonstrated greater sensitivity to delay or uncertainty in at least one task, suggesting that for these groups, reducing uncertainty in vaccine access, through guaranteed availability, clear timelines, and community-based distribution, may be more important than price reduction alone. SPTs offer a feasible tool for modeling these preferences before a vaccine becomes available, informing delivery strategies in advance of regulatory approval.

This study is among the first to apply behavioral economic discounting frameworks to HIV vaccine decision-making in an SGM sample, modeling constrained choices for a prevention technology that does not yet exist. The use of SPTs extends prior work on COVID-19 vaccine discounting [41] and healthcare wait-time valuation [79] to a novel and high-stakes public health context. Another strength of this research lies in its robust data collection from a population that researchers have noted to be traditionally challenging to engage [80]. This was possible through the use of alternative recruitment strategies, such as engaging participants through Reddit and leveraging community-based organizations serving SGMs. Although these strategies may introduce selection bias and limit generalizability, they likely increased access to individuals who are connected to SGM communities and willing to participate in online research, including uncompensated studies.

Although the present study offers valuable insights, several limitations should be considered when interpreting its findings. The use of non-probability convenience sampling through online platforms and community organizations introduces potential selection bias and limits generalizability [81]. Compounding this, recruitment links were not source-specific, preventing direct comparison of participants from Reddit versus community-based organizations. These groups may differ meaningfully in demographics, HIV risk, and decision-making patterns, and future studies should track recruitment sources to assess these differences. The study also lacked information on participants’ HIV status, precluding comparisons in vaccine valuation between HIV-positive and HIV-negative individuals. Because HIV status may substantially shape how vaccine benefits are perceived, this distinction remains an important area for future investigation. In addition, all participants completed both experiments in the same sequence, which may have introduced order effects [82]. Counterbalancing task order would strengthen internal validity in subsequent designs. Regarding measurement decisions, scenario parameters, including the $150 price point and effectiveness values were not formally pilot-tested. Although smooth aggregate discounting curves and high R² values suggest participants interpreted the scenarios consistently, incorporating formal comprehension checks would provide stronger confirmation in future research [83]. Vaccine acceptance was also assessed exclusively within the discounting task context. The tasks were specifically designed to elicit tradeoff-based responses under conditions of delay and uncertainty, which single global acceptance items do not capture and which may yield higher stated willingness than tradeoff-based tasks [84]. That said, a global measure administered alongside the tasks would have offered a useful comparison point, and we recommend including both in future studies. Similarly, while we assessed trust in science, medical professionals, and government, we did not directly measure general vaccine beliefs or vaccine-specific trust in science. These are conceptually distinct from attitudes toward vaccines specifically or toward an HIV vaccine in particular [85, 86], and future studies would benefit from including targeted vaccine belief measures. Finally, the single $150 price point limits conclusions about cost sensitivity. While this value approximated the median cost of existing vaccines at the time of study design [51], the actual out-of-pocket cost of a future HIV vaccine remains unknown and could differ substantially depending on insurance coverage, government subsidization, or public health distribution models. Future work should employ multiple price points to better characterize price-demand functions for HIV vaccines under different realistic cost scenarios.

Building on these limitations and measurement considerations, several avenues for future research could extend both the understanding and practical applicability of these findings. Replication in more diverse samples, including underrepresented risk groups such as, sex workers and people who inject drugs (PWIDs), is needed to determine whether subgroup differences observed here, particularly by age, insurance status, and sexual orientation, generalize beyond online-recruited SGM populations. Including participants from clinical and community-based healthcare settings would further strengthen external validity. Future research could also examine the substitutability and complementarity of HIV vaccines with other prevention tools such as PrEP, which would clarify how individuals weigh competing protective strategies and inform bundled intervention approaches. Finally, exploring additional conditions within delay and probability discounting tasks, such as manipulating price more parametrically using multiple price points, could reveal more nuanced understandings of how cost impacts vaccine valuation over time or under uncertainty. This would also allow for a more detailed analysis of price sensitivity and its role in vaccine acceptance.

## Data Availability

Available upon request.
